# The epidemiology of pediatric traumatic brain injury presenting at a referral center in Moshi, Tanzania

**DOI:** 10.1371/journal.pone.0273991

**Published:** 2022-10-05

**Authors:** Loren K. Barcenas, Roselyn Appenteng, Francis Sakita, Paige O’Leary, Henry Rice, Blandina T. Mmbaga, Joao Ricardo Nickenig Vissoci, Catherine A. Staton

**Affiliations:** 1 Duke Global Health Institute, Durham, NC, United States of America; 2 Vanderbilt University, Nashville, TN, United States of America; 3 Kilimanjaro Christian Medical Centre, Moshi, Tanzania; 4 Division of Emergency Medicine, Duke University School of Medicine, Durham, NC, United States of America; 5 Kilimanjaro Clinical Research Institute, Moshi, Tanzania; 6 Kilimanjaro Christian Medical University College, Moshi, Tanzania; Indiana University School of Medicine, UNITED STATES

## Abstract

**Background:**

Over 95% of childhood injury deaths occur in low- and middle-income countries (LMICs). Patients with severe traumatic brain injury (TBI) have twice the likelihood of dying in LMICs than in high-income countries (HICs). In Africa, TBI estimates are projected to increase to upwards of 14 million new cases in 2050; however, these estimates are based on sparse data, which underscores the need for robust injury surveillance systems. We aim to describe the clinical factors associated with morbidity and mortality in pediatric TBI at the Kilimanjaro Christian Medical Centre (KCMC) in Moshi, Tanzania to guide future prevention efforts.

**Methods:**

We conducted a secondary analysis of a TBI registry of all pediatric (0–18 years of age) TBI patients presenting to the KCMC emergency department (ED) between May 2013 and April 2014. The variables included demographics, acute treatment and diagnostics, Glasgow Coma Scores (GCSs, severe 3–8, moderate 9–13, and mild 14–15), morbidity at discharge as measured by the Glasgow Outcome Scale (GOS, worse functional status 1–3, better functional status 4–6), and mortality status at discharge. The analysis included descriptive statistics, bivariable analysis and multivariable logistic regression to report the predictors of mortality and morbidity. The variables used in the multivariable logistic regression were selected according to their clinical validity in predicting outcomes.

**Results:**

Of the total 419 pediatric TBI patients, 286 (69.3%) were male with an average age of 10.12 years (SD = 5.7). Road traffic injury (RTI) accounted for most TBIs (269, 64.4%), followed by falls (82, 19.62%). Of the 23 patients (5.58%) who had alcohol-involved injuries, most were male (3.6:1). Severe TBI occurred in 54 (13.0%) patients. In total, 90 (24.9%) patients underwent TBI surgery. Of the 21 (5.8%) patients who died, 11 (55.0%) had severe TBI, 6 (30.0%) had moderate TBI (GCS 9–13) and 3 (15.0%) presented with mild TBI (GCS>13). The variables most strongly associated with worse functional status included having severe TBI (OR = 9.45) and waiting on the surgery floor before being moved to the intensive care unit (ICU) (OR = 14.37).

**Conclusions:**

Most pediatric TBI patients were males who suffered RTIs or falls. Even among children under 18 years of age, alcohol was consumed by at least 5% of patients who suffered injuries, and more commonly among boys. Patients becoming unstable and having to be transferred from the surgery floor to the ICU could reflect poor risk identification in the ED or progression of injury severity. The next steps include designing interventions to reduce RTI, mitigate irresponsible alcohol use, and improve risk identification and stratification in the ED.

## Introduction

Childhood injuries account for nearly 10% of the total pediatric burden of disease worldwide, 84% of which are unintentional [1]. Among unintentional injuries, traumatic brain injuries (TBIs) [2] are the most frequently occurring injury in children under 15 years of age globally [1]. Global deaths due to injuries are projected to increase by 28% between 2004 and 2030 [3]. According to certain estimates, TBIs accounted for 82,000 deaths in Europe [4] and 50,000 deaths in the United States [5]. In Africa, TBI estimates are projected to increase up to 14 million by 2050 [6].

TBIs represent a significant health burden in the pediatric population globally; however, low- and middle-income countries (LMICs) account for 95% of childhood injury deaths [3]. Similarly, patients with severe TBI in LMICs have twice the likelihood of dying compared with their respective cohort in high-income countries (HICs) [7, 8]. TBIs are expected to surpass many diseases as a main cause of mortality and morbidity in LMICs by 2030 [9]. Yet, the available data on pediatric TBI and trauma care from LMICs are limited [10]. Primary sources of information for trauma injuries come from inaccurate sources, such as vital registration data and police records, if present at all. Hospital recordkeeping may not be consistent and many TBI cases never make it to the hospital. Mild TBI often goes undiagnosed, whereas patients with severe TBI may die before they reach the hospital [11].

Children have a larger head to body ratio compared to adults, placing them at a significantly increased risk for TBI for similar mechanisms of injury [12, 13]. Additionally, the vast difference in the developmental characteristics of neonates to adolescents and young adults implies that the mechanisms of injury will vary across this population [14]. Depending on the severity of injury, the data suggest that TBIs significantly impairs neurocognitive processes, including executive functioning (attention, processing) in the pediatric brain [15, 16]. The age at which the injury occurs matters, as young children who experience TBI injuries have pronounced developmental disadvantages compared to older counterparts [15, 17]. Further evidence suggests that TBIs can impair social cognition in adolescents, which has meaningful implications for the ability to process information, make inferences, and respond appropriately in a social context [18]. Persistent neurologic deficits can hamper development in pediatric patients with TBI, leading to negative consequences for learning, academic performance, and quality of life [19].

Tanzania, situated in East Africa, has an estimated population of 45 million people and an estimated average life expectancy of 61.8 years [20] In a report on the disease burden observed in ICUs across the four tertiary referral centers in Tanzania, Sawe et al. found that trauma (22.2%) was the main disease category. Additionally, across all age groups, intracranial injury (12.5%) was the leading diagnosis [21]. Previous data have been reported on the epidemiology of TBIs presenting at Kilimanjaro Christian Medical Centre (KCMC) in a registry as a quality improvement initiative [22, 23]. Trauma registries enable the comprehensive collection of data elements for subsequent analysis and the tracking of quality improvement initiatives.

However, previous data have largely focused on adult populations. Studying pediatric populations, specifically, is important as the mechanism of TBI varies widely across age groups [10]. In addition, age is significantly associated with differences in mortality rates between pediatric patients and adults and within pediatric populations [24, 25]. A lack of data describing TBI in youth populations limits the ability of physicians to provide the highest-quality care. This project aims to determine the clinical factors associated with morbidity and mortality from TBI in pediatric populations at KCMC to point out specific areas in need of quality improvement.

## Methods

### Ethical consideration

The Ethics Committee at KCMC and the Institutional Review Board of Duke University granted approval for the prospective TBI registry data collection and analysis.

### Study design

Our study was designed as a secondary data analysis of a de-identified cross-sectional TBI registry using the subset of patients under 18 years of age from KCMC over a 1-year period [22]. All participants (or participants’ parents or guardians) provided written informed consent authorizing the collection and use of the data in this research. Patients presenting with acute TBI within 24 hours of the primary injury were enrolled in the registry. Their initial management and hospitalization were directly observed by trained research nurses who collected the TBI registry data and completed the follow-up of each patient’s course to ascertain outcomes at discharge. The registry data were initially collected on paper then transferred to Research Electronic Data Capture (REDCap), a web-based database application [26].

### Setting

As one of the country’s four tertiary referral centers and the country’s third largest hospital, a significant number of TBI cases present to KCMC in Moshi, Tanzania. Regional data estimate that TBIs comprise 6% of annual Emergency Department (ED) visits, or about 1,000 patients. Over a 1-year period, 893 adult patients who presented acutely to KCMC were enrolled in the TBI registry, of which 12.9% were diagnosed with severe TBI, with a mortality rate of 47% [22].

### Study population

The KCMC TBI registry included all patients who presented to the KCMC ED for initial management of acute (<24 hours) TBI. Patients presenting for follow-up care, or non-acute injuries were excluded from the study, as well as those patients who did not survive to evaluation. This secondary analysis evaluated only the subset of patients who were 18 years of age or younger.

### Variables

The clinical TBI registry included variables on the patients’ demographics, injury characteristics, acute treatment, acute diagnostics, functional status, morbidity, and mortality. The most pertinent variables to our analysis are shown in Table 1. Airways were assessed as either intact or not intact. Whether or not patients could move their extremities or had seizure-like activity was assessed. Whether or not patients were treated with fluids, oxygen, a head CT scan, TBI surgery, or going to the ICU was assessed. Airway management, including intubation, nasal, or oral airway management, was dichotomized into performed or not performed. Hypoxia was defined as a pulse oxygenation of less than 92%. Blood pressure was categorized based on systolic blood pressure and age into [27] hypotension (ages ≤ 1: ≤ 75, ages 2–5: ≤ 80, ages 6–13: ≤ 90, ages 14–18: ≤ 105), normotension, (ages ≤ 1: 75–90, ages 2–5: 80–95, ages 6–13: 90–105, ages 14–18: 105–117) and hypertension (ages ≤ 1: > 90, ages 2–5: >95, ages 6–13: > 105, ages 14–18: >117). [27, 28] Level of consciousness was measured by the AVPU (Alert/Verbal/Pain /Unresponsive) scale, which categorized patients into alert, responsive to voice, responsive to pain, or unresponsive. Alcohol use was confirmed by clinical exam, breathalyzer test, or patient self-report of using alcohol within 6 hours of the injury. Injury severity was determined by the Glasgow Coma Scale (GCS): Severe injuries were scored as 0–8, moderate injuries were scored as 9–13, whereas mild injuries were scored as 14 or 15 on the GCS.

**Table 1:** Patient demographics and injury epidemiology.

		Original Data N = 419
**Age**	Age, Median (IQR)	10 (5;16)
	Missing, n (%)	0 (0)
**Gender**, n (%)	Male	286 (68.3)
	Female	127 (30.3)
	Missing	6 (1.4)
**Mechanism of Injury**, n (%)	Road Traffic Injury	269 (64.2)
	Fall	82 (19.6)
	Other	67 (16.0)
	Missing	1 (0.2)
**Alcohol Involvement**, n (%)	Alcohol involved	23 (5.5)
	Missing	22 (5.3)
**GCS**, n (%)	Mild	315 (75.2)
	Moderate	46 (11.0)
	Severe	54 (12.9)
	Missing	4 (1.0)
**Vitals**	Temperature, Median (IQR)	36.3 (36;37)
	Missing, n (%)	43 (10.3)
	Respiratory Rate, Median (IQR)	20 (20;24)
	Missing, n (%)	71 (16.9)
	Heart Rate, Median (IQR)	96 (80;194)
	Missing, n (%)	54 (12.9)
**Blood Pressure**, n (%)	Hypotensive	35 (8.4)
	Normotensive	60 (14.3)
	Hypertensive	139 (33.2)
	Missing	185 (44.2)
**Pulse Oxygen**, n (%)	Hypoxic	37 (8.8)
	Missing	81 (19.3)
**AVPU**, n (%)	Alert	321 (76.6)
	Responsive	68 (16.2)
	Unresponsive	14 (3.3)
	Missing	16 (3.8)
**Oxygen Applied**, n (%)	Oxygen Applied	15 (3.6)
	Missing	1 (0.2)
**Chest Radiograph**, n (%)	Chest Radiograph Received	35 (8.4)
	Missing	0 (0)
**Fluids Started**, n	Fluids Started	155 (37.0)
	Missing	3 (0.7)
**Labs Sent**, n	Labs Sent	346 (82.6)
	Missing	0 (0)
**Moved All Extremities**, n	Moved all extremities	372 (88.8)
	Missing	3 (0.7)
**Seizure-like Activity**, n	Seizure-like Activity	7 (1.7)
	Missing	3 (0.7)
**Skull Radiograph**, n	Skull Radiograph Received	330 (78.8)
	Missing	2 (0.5)
**CT Brain Scan**, n	CT Brain Scan Received	58 (13.8)
	Missing	1 (0.2)
**Mannitol**, n	Mannitol administered	21 (5.0)
	Missing	2 (0.5)
**Seizure Medication**, n	Seizure Medication Administered	330 (78.8)
	Missing	2 (0.5)
**TBI Surgery**, n	TBI Surgery Received	90 (21.5)
	Missing	57 (13.6)
**ICU**, n (%)	Went to ICU	55 (13.1)
	Missing	75 (17.9)

### Outcomes

Mortality and morbidity were determined based on the Glasgow Outcome Scale (GOS) or the Glasgow Outcome Scale Extended (GOSE), depending on which survey was administered to the patient. Initially, the GOS was adopted during the initial phases of the registry but given its inability to adequately define levels of disability among our population, we switched to the GOSE. For both the GOS and the GOSE, a score of 1 indicated death, or mortality. Those with a GOS of 1–3 or a GOSE of 1–4 was considered to have a worse functional outcome, whereas those with a GOS of 4–5 or a GOSE of 5–8 had a better functional outcome.

### Statistical analyses

Descriptive statistics were summarized using the mean and standard deviation for the continuous data. The categorical data were summarized using frequencies and percentages. Using a t-test, a chi-squared test, or Fisher’s exact test when appropriate, the bivariable analysis determined the magnitude and significance of the association of each predictor with mortality and morbidity individually using a P value cut-off of 0.001.

Data imputation was necessary to handle missing values in order to have non-zero values for variables with low counts to proceed with the statistical analysis.

Multivariable logistic regression models were fit to adjust for confounding in the association between the predictors and the outcomes, including variables according to theoretical validity. Backwards selection was used to show the most important clinical predictors associated with mortality and morbidity, adjusting for confounding factors. Statistical analysis of the data was performed using R, an open-source programming software.

## Results

### Patient demographics and injury epidemiology

**Fig 1:**
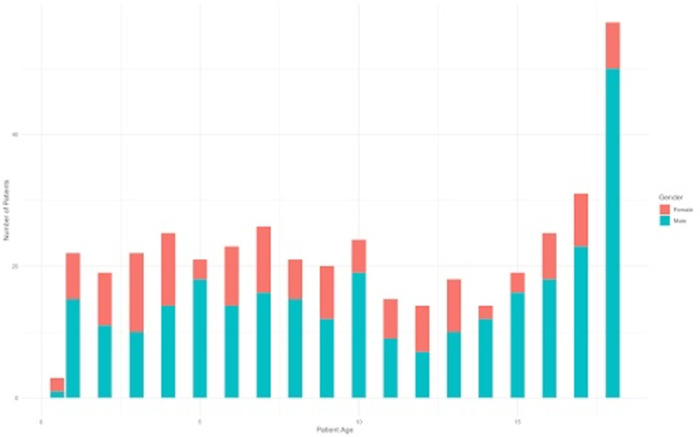
Pediatric patients by age and gender. The frequency of pediatric patients at each age broken down into the number of males and females of that age.

**Fig 2:**
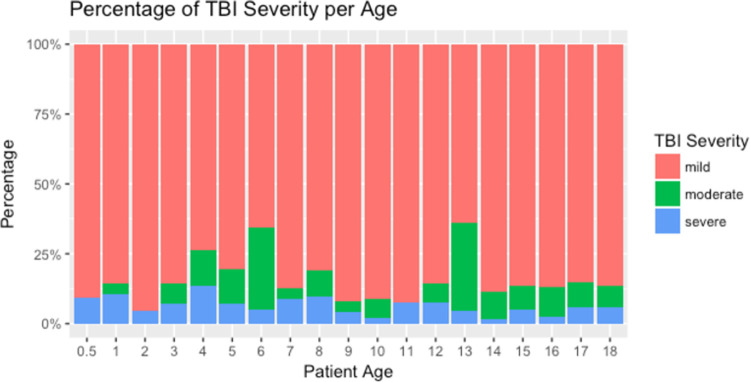
Pediatric patients by age and injury severity. The percentage of mild, moderate, and severe traumatic brain injury for pediatric patients at each age.

Between May 5, 2013 and April 25, 2014, a total of 419 pediatric patients ages 18 and under were enrolled in the TBI registry. Patient characteristics and frequency of missing data are described in Table 1. The median age was 10 years (IQR 5–16) and 68.7% were male. Fig 1 shows the distribution of patients by age and gender. We found the number of TBI patients for each age to be relatively even among children under 10 years of age (range = 19–26, median = 23), lower among 11- to 15-year-olds (range = 14–19, median = 15), rising frequency in 16- to 17-year-olds (range = 25–31, median = 28), and significantly higher in those 18 years of age (n = 57, 13.6%). The majority (74.7%) of TBI cases were mild (GCS 14–15), whereas 11% were moderate (GCS 9–13) and 12.6% were severe (GCS 3–8). While the majority of injuries were mild TBIs, more moderate and severe TBIs were observed in children 4–6 years of age and at 13 years of age (Fig 2). The most common mechanism of injury across all age groups was road traffic injury (RTI) at 64.4%, followed by falls at 19.8%. Although the legal drinking age of the region is 18 years of age, alcohol use was confirmed in 13 patients were under the age of 18, ranging from 5–17 years (median = 16 years), of which 69.2% (n = 9 of 13) were male.

### Emergency department disposition and outcomes

Overall, 21.5% (n = 90) pediatric patients underwent surgery for their TBI. Of those patients who underwent surgery, 10% (n = 9) died. The majority of patients were admitted to the surgical wards (82.8%), whereas few went directly to the ICU (3.1%) or the operating theatre (0.5%). Overall, 12.4% (n = 52) patients spent time on the surgery floor before being moved to the ICU. In total, 5.7% (n = 24) pediatric patients died and 6.4% (n = 27) had a worse functional status, defined as a GOS score of less than 4 (Table 2).

**Table 2:** Patient and injury characteristics by outcome.

			Mortality Median (IQR) or n (%)			Morbidity		
	Variable	Total N = 419	Alive n = 395	Dead n = 24	*P* value	Better Functional Status n = 392	Worse Functional Status n = 27	*P* value
	Age		10 (5;16)	12 (5.8;16.3)		10 (5;16)	12 (6;15.5)	
**Gender**	Male	291	278 (95.5)	13 (4.5)	0.15	276 (94.8)	15 (5.2)	0.16
	Female	128	117 (91.4)	11 (8.6)		116 (90.6)	12 (9.4)	
**Mechanism of Injury**	Road Traffic Injury	270	250 (92.6)	20 (7.4)	0.16	247 (91.5%)	23 (8.5)	0.09
	Fall	82	80 (97.6)	2 (2.4)		80 (97.6)	2 (2.4)	
	Other	67	65 (97)	2 (3)		65 (97)	2 (3)	
**Alcohol Involvement**	No	393	370 (94.1)	23 (5.9)	0.99	367 (93.4)	26 (6.6)	0.99
	Yes	26	25 (96.2)	1 (3.8)		25 (96.2)	1 (3.8)	
**GCS**	Mild	313	310 (99.0)	3 (1.0)	<0.001	310 (99.0)	3 (1.0)	<0.001
	Moderate	46	39 (84.8)	7 (15.2)		39 (84.8)	7 (15.2)	
	Severe	53	39 (73.6)	14 (26.4)		36 (67.9)	17 (32.1)	

### Airway/Breathing

Airway assessments were performed on all patients, and only 7 were found to be not intact. Of those, 1 was intubated and 5 had oral or nasal airway management. Oxygen was applied to 3.6% of all pediatric patients and 23.2% of those who were hypoxic (n = 13 of 56) with a pulse oxygenation of less than 92% (Table 3). Only 7.9% (n = 33) of patients were auscultated by the treatment team, and 8.1% (n = 34) received a chest radiograph either in the ED at KCMC or prior to arrival.

**Table 3:** Patient vital signs and overall presentation.

			Mortality			Morbidity		
	Variable	Total	Alive Mean (SD) or n (%)	Dead Mean (SD) or n (%)	P value	Better Functional Status	Worse Functional Status	P value
Vitals	Temperature		36.5 (0.8)	36.5 (1.3)	0.82	36.5 (0.8)	36.5 (1.2)	0.16
	Respiratory Rate		23.2 (4.5)	22.7 (5.1)	0.65	23.2 (4.5)	23.3 (5.8)	0.94
	Heart Rate		93.7 (19.2)	98.8 (32.8)	0.49	93.9 (19.2)	95.6 (31.8)	0.80
Blood Pressure	Hypotensive	46	2 (4.3)	41 (89.1)	0.91	44 (95.7)	2 (4.3)	0.86
	Normotensive	87	4 (4.6)	72 (82.8)		81 (93.1)	5 (5.7)	
	Hypertensive	286	15 (5.2)	231 (80.8)		262 (91.6)	20 (7.0)	
Pulse Oxygen	Hypoxic	56	43 (76.8)	11 (19.6)	0.01	41 (73.2)	13 (23.2)	0.01
	Non-hypoxic	363	347 (95.6)	13 (3.6)		346 (95.3)	14 (3.9)	
AVPU	Alert	319	317 (99.4)	2 (0.6)	<0.001	317 (99.4)	2 (0.6)	<0.001
	Responsive	78	65 (83.3)	14 (17.9)		62 (79.5)	16 (20.5)	
	Unresponsive	14	6 (42.9)	8 (57.1)		6 (42.9)	8 (57.1)	

### Circulation/Deficit

Fluids were given to 36.5% (n = 153 of 419) of all pediatric patients, but of those who were hypotensive (by age) only 14.4% received fluids (n = 22 of 153). Fluids were given to 64.7% of those who had a non-TBI related surgery, while 37.8% who did not have any surgery received fluids. Lab work was ordered and sent for 81.4% of all pediatric patients (n = 341 of 419) and 100% of those who were hypotensive (n = 38). Treatment teams observed seizure-like activity among 1.7% (n = 7) patients, of which 57% (n = 4 of 7) received anti-seizure medication either prior to arrival or in the ED at KCMC. Head CT scans were performed on 13.4% of patients (n = 56), and skull radiographs were performed on 71.6% of patients (n = 300). Mannitol was administered to 4.8% of patients (n = 20) (Table 4).

**Table 4:** Treatments and diagnostics.

			Mortality			Morbidity		
	Variable	Total	Alive Mean (SD) or n (%)	Dead Mean (SD) or n (%)	P value	Better Functional Status	Worse Functional Status	P value
Oxygen Applied	No	398	382 (96.0)	16 (4.0)	<0.001	380 (95.5)	18 (4.5)	<0.001
	Yes	15	7 (46.7)	8 (53.3)		6 (40.0)	9 (60.0)	
Chest Radiograph	No	379	356 (93.9)	23 (6.1)	0.73	353 (93.1)	26 (6.9)	0.73
	Yes	34	33 (97.1)	1 (2.9)		33 (97.1)	1 (2.9)	
Fluids Started	No	259	253 (97.7)	6 (2.3)	<0.001	253 (97.7)	6 (2.3)	<0.001
	Yes	153	135 (88.2)	18 (11.8)		132 (86.3)	21 (13.7)	
Labs Sent	No	73	71 (97.3)	2 (2.7)	0.28	71 (97.3)	2 (2.7)	0.20
	Yes	341	319 (%)	22 (6.5)		316 (92.7)	25 (7.3)	
Moved All Extremities	No	42	29 (69.0)	13 (31.0)	<0.001	28 (66.7)	14 (33.3)	<0.001
	Yes	370	359 (97)	11 (3.0)		357 (96.5)	13 (3.5)	
Seizure-like Activity	No	405	383 (94.6)	22 (5.4)	0.08	380 (93.8)	25 (6.2)	0.11
	Yes	7	5 (71.4)	2 (28.6)		5 (71.4)	2 (28.6)	
Skull Radiograph	No	85	74 (87.1)	11 (12.9)	0.01	74 (87.1)	11 (12.9)	0.02
	Yes	300	287 (95.7)	13 (4.3)		284 (94.7)	16 (5.3)	
CT Brain	No	358	341 (95.3)	17 (47.5)	0.03	338 (94.4)	20 (5.6)	0.06
	Yes	56	49 (87.5)	7 (12.5)		49 (87.5)	7 (12.5)	
Mannitol	No	394	373 (94.7)	21 (5.3)	0.10	370 (93.9)	24 (6.1)	0.13
	Yes	20	17 (85.0)	3 (15.0)		17 (85.0)	3 (15.0)	
Seizure Medication Administration	No	405	383 (94.6)	22 (5.4)	<0.001	380 (93.8)	25 (6.2)	<0.001
	Yes	4	1 (25.0)	3 (75.0)		1 (25.0)	3 (75.0)	
TBI Surgery	No	271	260 (95.9)	11 (4.1)	0.06	260 (95.9)	11 (4.1)	<0.001
	Yes	88	79 (89.8)	9 (10.2)		76 (86.4%)	12 (13.6)	
ICU	No	287	285 (99.3)	2 (0.7)	<0.001	285 (99.3)	2 (0.7)	<0.001
	Yes	52	39 (75.0)	13 (25.0)		37 (71.2)	15 (28.8)	

### Predictors of worse outcomes

**Fig 3:**
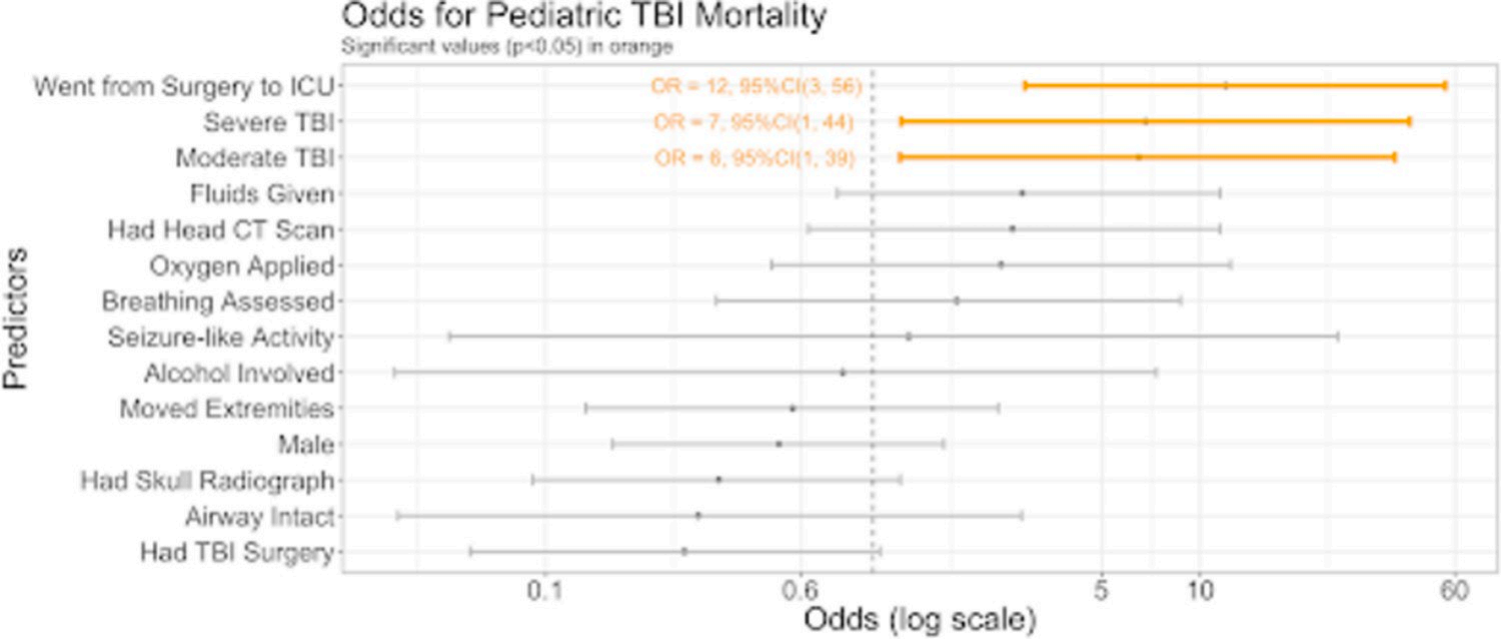
Odds of mortality. The odds (on a log scale) of mortality based on multivariable logistic regression for the pediatric TBI patients by each predictor variable.

**Fig 4:**
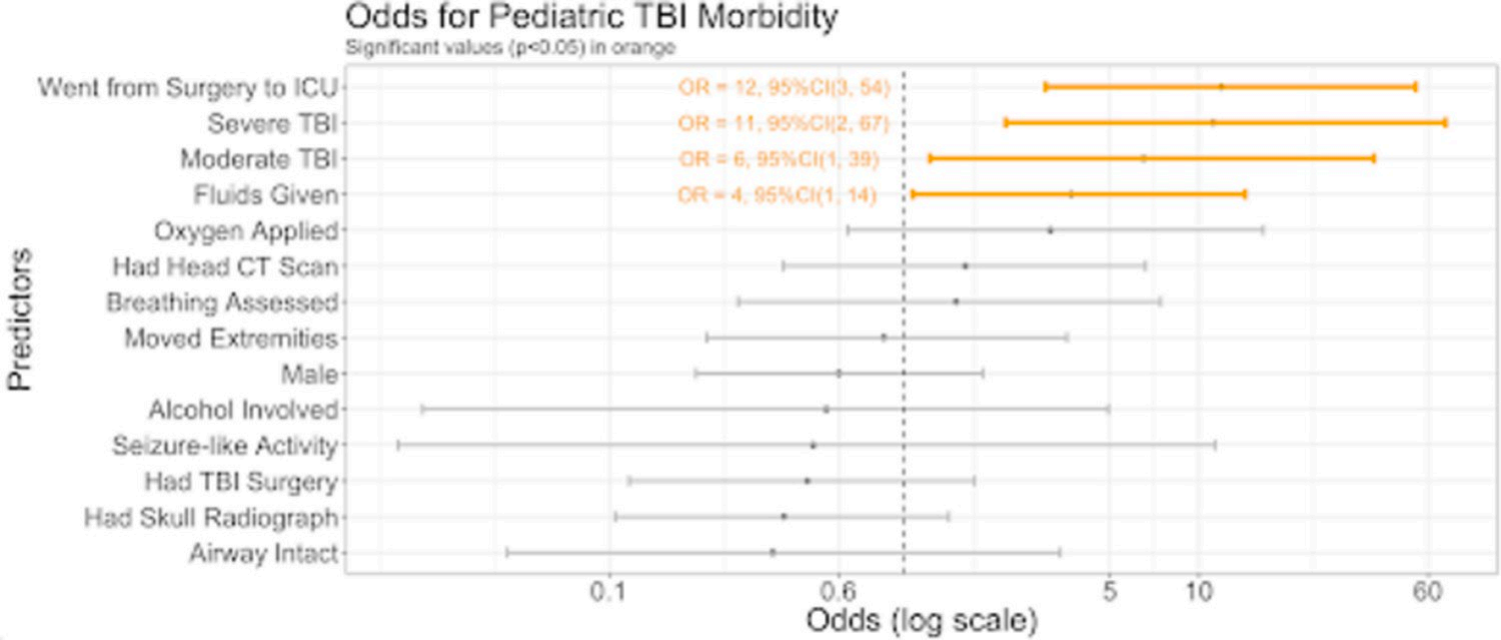
Odds of morbidity. The odds (on a log scale) of morbidity based on multivariable logistic regression for the pediatric TBI patients by each predictor variable.

Multivariable regression analysis revealed that being moved from the surgery floor to ICU (OR = 12, 95% CI 3,56), having severe (OR = 7, 95% CI 1,44) or moderate (OR = 6, 95% CI 1,39) TBI severity were significantly associated with mortality (Fig 3). The variables associated morbidity also included being moved from the surgery floor to the ICU (OR = 12, 95% CI 3,54), severe (OR = 11 95% CI 2,67), and moderate (OR = 6, 95% CI 1,39) TBI severity, as well as receiving fluids (OR = 4, 95% CI 1,14) as seen in Fig 4.

Bivariable analysis, as reported in Tables 2–4, shows following variables appeared statistically significant: GCS, AVPU, oxygen applied, fluids given, moved all extremities, seizure medication administration, TBI surgery (morbidity only), and ICU. Out of these variables, there was overlap between the statistical significance with the multivariable model with GCS, fluids given (morbidity only), and ICU.

## Discussion

This is the first study to attempt to identify predictors of mortality and morbidity from TBI within a pediatric population in Tanzania. The study was based on a large prospective clinical TBI registry in Tanzania. Understanding the unique characteristics associated with TBI can help inform targeted prevention efforts, improve clinical guidelines, and increase the quality of care. At KCMC a significant number of patients deteriorated in the surgery ward, and patients that received IV fluids were associated with worse functional status.

### Epidemiology

Within the pediatric TBI population at KCMC, we found a male-to-female ratio of about 2:1. Our findings are relatively consistent with the literature in Sub-Saharan Africa, which reports an average male-to-female ratio of 3:1 [29–34]. Males may be more prone to risky behaviors that lead to injuries possibly due to gender roles [35].

We found the distribution of TBI to be relatively even among children under 10 years of age, lower among 11- to 15-year-olds, and significantly increased in those 18 years of age. This distribution differs from the literature, which typically cites a bimodal distribution of TBI in those under 2 years of age and adolescents (age 15–18) globally [36]. Other studies have shown that assault is one of the main causes of TBI, leading to hospitalization and death in infants globally [37, 38] however, our data do not reflect this, and instead indicates that falls and RTIs contribute to more TBIs. Survivor bias may have lowered our sample’s rates of TBIs in those under 2 years of age due to long transport times created by the tertiary referral status of our hospital.

Overall, more than 5% of our sample suffered alcohol-related injuries, half of which were under the legal drinking age (the majority being 17 years old). This rate is similar to the percentages of pediatric alcohol-related injuries generally seen in both LMICs and HICs. In Southern Taiwan, approximately 7.7% of pediatric trauma patients tested positive for blood alcohol concentration [39]. Another study in Queensland, Australia focusing on patients 12–24 years of age found that 6.4% of injuries were alcohol related [40]. Similarly, alcohol use is a significant problem among adolescents in Northern Tanzania [41, 42]. High exposure to alcohol advertisements and wide availability of alcohol products contribute to alcohol consumption among youth in Northern Tanzania [42, 43]. However, studies across Africa have shown that parental disapproval and monitoring of drinking are protective factors against alcohol uptake in adolescents [44]. Further evaluation of alcohol among this adolescent group is needed, especially in the setting of harm reduction for this at-risk youth group.

RTI was by far the most common mechanism of injury, accounting for 64.4% of all patients in this study, which is comparable to global estimates. Reported percentages of RTI in children with TBI range from 48.3% in the Royal London Hospital to 53.3% in a Nigerian tertiary hospital and 60.7% in a tertiary center in Malawi [34, 45]. One study estimating the global incidence of TBI found that the overall proportion of TBI resulting from road traffic crashes (RTCs) to be approximately 56% [10]. RTIs are the ninth leading cause of death in the world and the leading cause of TBI in the pediatric population [3] Typically following urbanization, a surge occurs in the use of vehicles, which leads to an increased number of RTCs, making RTI a major concern in developing LMIC settings [2, 9, 10]. RTIs include not only injured drivers and passengers, but also pedestrians injured by motor vehicles. A study in Southwestern Uganda found that the majority of pediatric RTIs occur in unsupervised children who are pedestrians [31]. Further studies should be performed to determine areas of intervention and to identify specific risks kids are most vulnerable to.

### Predictors of outcome

The predictors of mortality in our study population based on the multivariable model were moderate and severe TBI (defined as a GCS of 3–8 and 9–13, respectively), making GCS an important predictor of death. Additionally, as expected, severe and moderate TBI were predictors of worse functional status, as patients with a lower GCS have more disabilities than those with a higher GCS. Other predictors of worse functional status include being transferred from the surgery floor to the ICU and receiving IV fluid therapy.

Those who were transferred from the surgery floor to the ICU were shown to be at a significantly higher risk for worse functional status than the patients who went straight to the ICU. This implies that either a subset of patients was initially assessed at admission to the emergency department (ED) as stable enough for surgery were mis-triaged, or their condition unexpectedly worsened enough to warrant transferring them to the ICU. Similarly, when analyzing the entire TBI registry 76.9% of patients who died in the ICU had been transferred from ED, compared to 23.1% who died after going straight to the ICU [22]. Deterioration during hospitalization can happen, Lee et al. in China found that 1.5% of adult patients with mild closed head injury deteriorated within seven days of the initial head injury, mostly due to intracranial hematomas [46].

Intravenous fluid therapy was associated with an increased risk of worse functional status, with an odds ratio of nearly 4. In the setting of TBI, fluids are typically given for insensible losses and hypovolemia from trauma co-occurring with TBI [47]. Fluid management in pediatric trauma is complex especially in LMIC and in limited monitoring settings.

One tool used to monitor and evaluate TBI patients at KCMC is skull radiography. Within this pediatric population of TBI patients, those who received a skull radiograph had better outcomes in mortality and morbidity compared to those who did not, although it is not clear why. Skull radiography can be used in trauma imaging in the absence of available CT scans to investigate the skull vault and associated bony structures. However, it is seldom used in HICs as the extent, type, and treatment plan for potential intracranial hemorrhages would remain undelineated [48]. Nonetheless, at KCMC this is the tool available to trauma patients for detecting injuries to the head and our data shows this imaging is associated with a lower morbidity and mortality.

During the time period of this study, the care for pediatric TBI patients was not protocolized and there were no trained EM physicians employed. Deciding which patients should receive tomographic scans is difficult when there is a lack of guidance appropriate for this setting. One way to provide diagnosis and treatment guidance for adolescents with mild TBI is through the use of clinical decision aids and clinical practice guidelines. However, these are not typically implemented or validated in LMICs. Alternatively, some LMICs have improved outcomes by developing their own protocols for trauma management [49–52]. The African Federation of Emergency Medicine (AFEM) and the WHO have available guidelines on the appropriate resources needed for care of pediatric trauma patients. These guidelines are adjustable based on local resources. By adjusting the guidelines to specific hospitals this creates a locally developed standardized trauma protocol (STPs). STPs have been found to be effective in improving outcomes. Specifically, in a level 1 trauma center in Colombia, the use of a STP resulted in an increased use of timely interventions for trauma patients, a decrease in discharge Glasgow Coma Scores and a decrease in mortality rates particularly amongst those with severe TBIs [53].

As such, the management of TBI is a dynamic process requiring continuous monitoring; limited reassessment, delays in treatment or inappropriate triage during this dynamic process may negatively impact outcomes. However, our datasets currently do not have enough data to assess the causes or processes associated with these patients’ deterioration. As such, further study into the risk factors for deterioration in patients with moderate TBI is needed. Similarly, improving risk identification and stratification in the Emergency Department to more easily recognize and prevent a patient from destabilizing is needed.

### Limitations

Our study was limited by the low prevalence of worse functional status within the sample, making it difficult to detect accurate statistical associations within this sub-group. Even though we had an overall sample size of 419 patients, the majority did not experience mortality or morbidity, limiting our power to detect an association. We had missing data, which reflects the difficulty of collecting clinical data in this setting. We compensated for missing data using data imputation. Likewise, our sample is affected by survivor bias as those who do not make it to the hospital are not represented. In this region which lacks pre-hospital care, pediatric patients may suffer from airway problems before reaching the hospital. Our data reflects the hospital-based mortality rather than all the deaths in the region.

## Conclusions

Among the pediatric population at KCMC, TBI is most common among males and predominantly caused by RTCs. Alcohol-related injuries remain prevalent among youth despite this population being below the legal drinking age. To improve the quality of care at KCMC, the risk factors for deterioration among patients with moderate TBI must be identified. This study addressed a key research gap by identifying factors associated with morbidity and mortality from TBI in pediatric populations and highlights the importance of studying TBI amongst pediatric populations in low income settings to improve care.
